# Prenatal Exposure to Cigarette Smoke Induces Diet- and Sex-Dependent Dyslipidemia and Weight Gain in Adult Murine Offspring

**DOI:** 10.1289/ehp.0800193

**Published:** 2009-04-13

**Authors:** Sheung P. Ng, Daniel J. Conklin, Aruni Bhatnagar, Duane D. Bolanowski, Jessica Lyon, Judith T. Zelikoff

**Affiliations:** 1 New York University School of Medicine, Nelson Institute of Environmental Medicine, Tuxedo, New York, USA; 2 University of Louisville, Institute of Molecular Cardiology, Louisville, Kentucky, USA

**Keywords:** cardiovascular disease, cigarette smoke, dyslipidemia, fetal basis of adult disease, fetal insult, prenatal exposure, weight gain

## Abstract

**Background:**

Cardiovascular disease (CVD) affects 71 million American adults and remains the leading cause of death in the United States and Europe. Despite studies that suggest that the development of CVD may be linked to intrauterine growth or early events in childhood, little direct experimental evidence supports the notion.

**Objective:**

We investigated whether exposure to cigarette smoke *in utero* alters the risk of developing CVD later in life.

**Methods:**

We exposed B_6_C_3_F_1_ mice (via whole-body inhalation) to either filtered air or mainstream cigarette smoke (MCS, at a particle concentration of 15 mg/m^3^) from gestational day 4 to parturition. Adult offspring were fed a normal chow diet or switched to a high-fat diet 2 weeks before sacrifice. We measured dam and offspring body weight, plasma lipid parameters, lipoprotein subclass particle numbers and sizes, and total antioxidant capacities.

**Results:**

Adult female mice prenatally exposed to MCS demonstrated significantly higher body weight and levels of plasma high-density lipoprotein (HDL) and low-density lipoprotein than did their air-exposed counterparts. When fed a high-fat diet for 2 weeks, males, but not females, exposed prenatally to MCS gained substantially more weight and exhibited dramatic alterations in total cholesterol and HDL levels compared with their air-exposed counterparts.

**Conclusions:**

These data provide, for the first time, direct experimental evidence supporting the notion that prenatal exposure to cigarette smoke affects offspring weight gain and induces a lipid profile that could alter the offspring’s risk of developing CVD later in life.

Cardiovascular disease (CVD) leading to acute myocardial infarction, heart failure, and stroke affects 71 million American adults and remains the leading cause of death in the United States and Europe (American Heart Association 2006). Although adult lifestyle choices, nutritional habits, and levels of physical activity significantly affect CVD risk, a number of studies suggest that the development of CVD may be linked to intrauterine growth or early events in childhood (Barker 2000; Cohen 2004; Louey and Thornburg 2005). Despite a strong relationship between maternal smoking and birth defects, 13% of the 6 million American women who become pregnant each year continue to smoke throughout their pregnancy (Albrecht et al. 2004; March of Dimes 2000). This is particularly distressing because some cardiotoxic cigarette smoke (CS) constituents [e.g., benzo(*a*) pyrene (BaP)] can cross the placental barrier (Perera et al. 2004). For example, in one study using atherosclerosisprone apolipoprotein E–null (ApoE^−/−^) mice, chronic oral exposure to BaP enhanced atherogenesis, producing larger lipid core plaques with higher levels of T lymphocytes than plaques formed in control animals without BaP exposure (Curfs et al. 2004). Because studies have shown that CD40 (a receptor present on macrophage and antigen-presenting cells) and its ligand CD40L (found on T lymphocytes) are associated with rupture-prone atherosclerotic plaques, an increase in lymphocyte numbers is of particular significance (Lutgens and Daemen 2002).

CS contains numerous chemical oxidants that contribute to inflammation and atheroclerotic plaque initiation and progression in exposed individuals (Link et al. 2007). Pryor and Stone (1993) reported that each puff of CS contains about 10^17^ oxidant molecules. Oxidization of low-density lipoprotein (LDL) particles is potentiated by oxidized free radicals in macrophages, leading to intracellular accumulation of LDL and the production of foam cells (Link et al. 2007). High-density lipoprotein (HDL) inhibits the oxidative modification of LDL and is anti-atherogenic based on its antiinflammatory properties and role in reverse cholesterol transport. The reverse cholesterol transport allows excess cholesterol in the periphery and in the atherosclerotic plaques of arterial walls to be transported to the liver and excreted from the body as bile salts (Link et al. 2007).

In several studies, lipid oxidation was linked to cardiac disease and atherosclerosis, the primary cause of heart disease (Gniwotta et al. 1997; Holvoet et al. 1998; Kovacs et al. 1997; Kummerow et al. 2000; Mosca et al. 1997). Kummerow et al. (2000) demonstrated that circulating levels of lipid oxidation products correlated with the degree of coronary artery stenosis in patients scheduled to receive cardiac catheterization. Furthermore, levels of F_2_-isoprostanes (products of lipid oxidation) have been found to be significantly increased in human atherosclerotic plaques compared with normal vascular tissue, supporting a role for oxidized lipids in the development of CVD (Gniwotta et al. 1997). Studies have also indicated that sex can play a role in the lipid-related development of CVD; women with coronary artery disease have been observed to have significantly greater levels of LDL lipid peroxidation (assessed by measurement of conjugated dienes and thiobarbituric acid-reactive substances) compared with men with the same disease (Mosca et al. 1997). This sexual dimorphism may be related to the fact that women have a higher overall risk of death from CVD and a poorer prognosis after myocardial infarction (Mosca et al. 2007).

Diet, as related to the intake of saturated fat and cholesterol, has been shown in a number of epidemiologic studies to be an important risk factor in the development of atherosclerosis (Kuller 2008). For example, in the Nurses’ Health Study (Hu et al. 1997), which involved 80,082 women (34–59 years of age) with no known history of coronary disease, stroke, cancer, hypercholesterolemia, or diabetes, results demonstrated that each 5% increase of energy intake from saturated fat (compared with equivalent energy intake from carbohydrates) was associated with a trend (relative risk = 1.17, *p* = 0.10) of increase in the risk of coronary disease. For a 2% increment in energy intake from *trans*-unsaturated fat (compared with equivalent energy from carbohydrates), the relative risk of developing coronary heart disease increased to 1.93 (*p* < 0.001); replacing saturated and *trans*-unsaturated fats with unhydrogenated monounsaturated and poly unsaturated fats was effective in preventing coronary heart disease.

CS-induced risk of CVD appears not to be limited to the smoker (Asmussen and Kjeldsen 1975; Berrahmoune et al. 2006; Hutchison et al. 1998; Noakes et al. 2007; Yang et al. 2007). Some early studies examining the effects of gestational CS exposure have revealed increased atherogenic changes in the umbilical cords of children from mothers who smoked during pregnancy (Asmussen and Kjeldsen 1975). Hutchison et al. (1998) demon strated that 4-week-old rats exposed to sidestream smoke *in utero* had increased aortic ring sensitivity to phenylephrine-induced vasoconstriction and reduced maximum endothelium-dependent acetylcholine-induced relaxation. These *ex vivo* studies suggested that early exposure to CS had detrimental effects on offspring vascular function. Moreover, recent studies in ApoE^−/−^ mice have demonstrated increases in atherosclerotic lesions and atherosclerosis-associated cytokine levels (i.e., monocyte chemoattractant protein-1 and tumor necrosis factor-α) in adult offspring of mothers exposed during pregnancy to environmental tobacco smoke (Berrahmoune et al. 2006; Yang et al. 2007). In humans, F_2_-isoprostanes, markers of lipid peroxidation, were measurably greater in the urine of 3-month-old infants after prenatal and postnatal CS exposure compared with infants of nonsmoking mothers (Noakes et al. 2007). Given the toxicologic and human studies relating prenatal and early postnatal CS exposure with lipid oxidation and subsequent CVD, these findings suggest an earlier onset of risk than previously expected.

CS also appears to act as a risk factor for CVD in prenatally exposed offspring by affecting fetal growth. In 1989, Barker et al. (1989) first proposed weight in infancy as an important and under recognized risk factor for CVD in later life. Since then, several studies have provided supporting evidence. For example, in an epidemiologic study, Martyn et al. (1998) revealed that the prevalence and severity of carotid atherosclerosis (assessed in carotid and lower-limb arteries) among 322 singleton infants born in the United Kingdom between 1922 and 1926 were greatest in those with the lowest recorded birth weight (after adjustment for other cardiovascular risk factors and gestational age at birth). In a study measuring aortic wall thickness (a marker of early atherosclerosis) and lipid profile, maximum and mean aortic intimamedia thickness and serum triglyceride (TRI) levels were significantly higher in babies with intrauterine growth retardation (*n* = 40) than in controls (*n* = 40) (Koklu et al. 2006). Thus, several mechanisms appear to exist by which prenatal CS exposure might act to predispose the offspring to increased CVD later in life.

Because increased risk for CVD can begin as early as the fetal/neonatal period after tobacco smoke exposure, we examined the subsequent effect(s) of fetal exposure to CS on offspring plasma lipoprotein levels and body weight (BW), two of the most significant CVD risk factors, as well as investigating whether, and how, exposure of the offspring to an additional risk factor [i.e., high-fat diet (HFD)] could affect these outcomes.

## Materials and Methods

### Animals, mating, and gestation

We housed 9- to 11-week-old male and female B_6_C_3_F_1_ mice (Jackson Laboratory, Bar Harbor, ME) in pairs (females) or individually (males) in polycarbonate cages with corncob bedding in temperature- (20–23°C) and humidity-controlled (55% relative humidity) rooms. During mating, pregnancy, and lactation, mice were provided food (LabDiet Mouse Diet 9F; PMI Nutrition International, LLC, Brentwood, MO) and tap water *ad libitum* unless otherwise specified. The light/dark cycle was maintained on 12-hr intervals. After acclimation for 1 week, mice weighing ≥ 20 g were paired. A single male mouse was paired with two females [considered gestational day (GD) 0]. After 4 days of pairing, the males were removed and the females were exposed (via whole-body inhalation) to either mainstream cigarette smoke (MCS) or filtered air (4 hr/day, 5 days/week) until parturition. At 3 weeks of age, offspring were weaned as described previously (Ng et al. 2006). All animals were treated humanely and with regard for alleviation of suffering, and all procedures with mice were approved by New York University Institutional Animal Care and Use Committee.

### Experimental design

We randomly separated 40 paired female mice (10–12 weeks of age) into two predesignated groups for exposure to either MCS or filtered air. Eight dams were selected from each exposure group on GD17, food deprived for 12 hr, weighed, and then sacrificed on GD18. Blood, collected by cardiac puncture using EDTA-coated 1-mL syringes (with 25-Ga needles), was mixed with 20 μL EDTA (0.2 M in distilled H_2_O) in Eppendorf tubes. Plasma (50 μL) samples were shipped immediately (at 4°C) to the Institute of Molecular Cardiology for analyses of total cholesterol (CHO), HDL, LDL, TRI, phospholipids (PPL), albumin (ALB), and total protein (TP). In addition, selected organs (i.e., hearts, livers, lungs, and kidneys) from dams in each treatment group were weighed and snap frozen in liquid nitrogen for future use.

Dams not sacrificed on GD18 were allowed to give birth (~ GD 21–22). After weaning, 16 offspring per sex from each exposure group (each pup from a different litter) were fed a normal chow diet [Laboratory Rodent Diet 5001 (13% calories provided by fat); PMI Nutrition International] until they were sacrificed at 13–14 weeks of age. In another group of animals at about the same age, we changed the diet of eight offspring per sex from each exposure group from normal chow to an HFD [TD.88137 Adjusted Calories Diet (42% from fat); Harlan Teklad, Madison, WI] 2 weeks before sacrifice. At the time of sacrifice (following a 12-hr fast), animals were weighed, blood was collected, and freshly collected plasma was shipped to the University of Louisville for analyses; selected organs (i.e., hearts, livers, kidneys) from all groups of offspring were weighed and snap frozen for future use. The remaining plasma from offspring fed a normal diet (three off-spring per sex from each exposure group) was snap-frozen in liquid N_2_ and shipped to the University of Louisville in dry ice. There, a portion (5 μL) of the plasma was used to measure total antioxidant capacity (TAC) (Erel 2004). The remainder (100 μL) of the plasma was then shipped overnight on dry ice to LipoScience, Inc. (Raleigh, NC) for determination of particle numbers and sizes of HDL, LDL, and VLDL (very low-density lipoprotein) subclasses using the nuclear magnetic resonance (NMR) LipoProfile test (Hammad et al. 2003).

### Smoke generation and exposure

We generated MCS from the burning of filtered 1R3F cigarettes (Kentucky Tobacco Research & Development Center, Lexington, KY) using an automated CS generation system (Baumgartner-Jaeger CSM 2070; CH Technologies Inc., Westwood, NJ). We generated smoke and exposed animals exactly as described previously (Ng et al. 2006). Mean ± SE chamber levels of carbon monoxide and total suspended particulates (TSP) were 23.4 ± 0.3 ppm and 17.1 ± 0.6 mg/m^3^, respectively. Taking into account chamber TSP levels, exposure duration, mouse BW, minute ventilation, and total particulate matter released from the burning of a single cigarette, this exposure level was roughly equivalent to smoking about one pack of cigarettes per day.

### Plasma lipid analyses

We measured concentrations of CHO, ALB, TP (reagents used for analyses purchased from Thermo Scientific, Waltham, MA), HDL, LDL, TRI, and PPL (reagents from Wako, Osaka, Japan) in 200-μL aliquots of plasma within 2 days of collection using a Cobas Mira Plus automated chemistry analyzer (Roche Diagnostic Systems, Inc., Branchburg, NJ).

### TAC assay

We measured TAC for the recovered plasma using the ABTS (2,2′-azinobis[3-ethylbenz-thiazoline-6-sulfonic acid]) method (Erel 2004). Briefly, 60 mL acetic acid solution (2.28% by volume) was added to 940 mL of a 400 mM sodium acetate (CH_3_COONa) solution (pH 5.8) and then stored at 4°C until needed (reagent 1). The second reagent was prepared by diluting 75 mL sodium acetate (30 mM) with 30 mM acetic acid and brought to a final volume of 1,000 mL (pH 3.6). We then used this solution to dilute 0.278 mL of 35% H_2_O_2_ to 1,000 mL, and used 100 mL of the resulting solution to dissolve 0.549 g ABTS for a final ABTS concentration of 10 mM (reagent 2). The resulting ABTS solution was then incubated at room temperature for 60 min until the appearance of the characteristic deep green color. On the day of the experiment, 0.2 mL reagent 1, 0.02 mL reagent 2, and 0.005 mL of the test sample were combined and incubated for 5 min; we determined absorbance (660 nm) before, during, and at the end of the incubation period. In some cases, we converted absorbance change to milli molar Trolox equivalents from a standard curve (0–1.5 mM).

### Statistical analyses

All values represent the mean ± SE. Sample sizes for each treatment group are given in figures and tables. In all cases, plasma samples were run in singlet using standard operating procedures. We used two-sample equal-variance *t*-tests to determine differences in lipid parameters and organ weights between MCS- and air-exposed dams. In both normal chow- and HFD-fed offspring groups, treatment differences (within a given sex) in lipid parameters and organ weights were determined by one-way analysis of variance (ANOVA) with post hoc testing (Fisher’s least significant difference analysis) when appropriate (SPSS, version 13.0; SPSS Inc., Chicago, IL). Because we wished to compare individual categories (e.g., smoke vs. air within each given sex), we regarded the four categories as the four levels of a single factor in a one-way ANOVA to be followed by post hoc comparisons when appropriate. Differences were considered significant at *p* < 0.05.

## Results

### Body and organ weights

**Figure 1:**
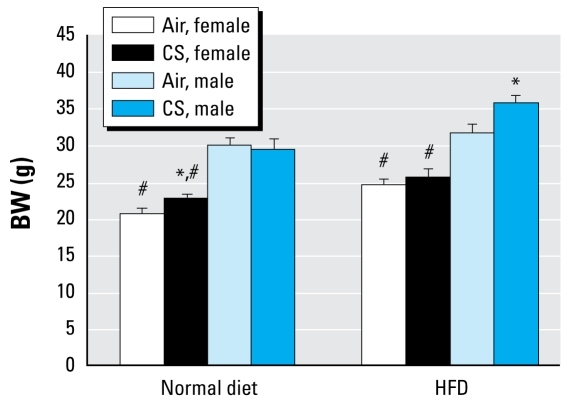
Effects of prenatal MCS exposure on BW of 13- to 14-week-old female and male offspring fed a normal diet or an HFD. Values represent the mean (*n* = 7–8 pups/sex/exposure regime/diet group) ± SE. *Significantly different (*p* < 0.05) from sex-matched, air-exposed offspring. ^#^Significantly different (*p* < 0.05) from exposure-matched male counterparts.

We determined BW along with weights of the heart, liver, kidneys, and lungs for both dams and offspring at the time of sacrifice. Inhalation exposure to MCS had no effect on dam BW or organ weights (Table 1). However, adult female pups pre natally exposed to MCS and fed a normal diet weighed significantly more than did female offspring born to air-exposed dams; we observed no differences in BW of adult male offspring from air- or MCS-exposed dams (Figure 1). Male offspring from both exposure groups weighed significantly more than their exposure-matched female counter parts.

When fed an HFD for 2 weeks, offspring of both sexes from air- and MCS-exposed dams gained weight (Figure 1). However, males exposed prenatally to MCS gained substantially more weight than their sex-matched air-exposed counterparts. These data revealed that *in utero* MCS exposure led to a higher basal BW in female offspring, whereas male offspring gained more weight on an HFD, an important risk factor for CVD.

Not surprisingly, we observed significant differences between the sexes in some organ weight measurements (Table 2). Absolute weights of the heart, liver, and kidney were lower in the female offspring (from both air- and MCS-exposed groups) than in the exposure-matched males. In contrast, females from both exposure groups had a greater heart weight/BW ratio than their male counterparts; relative liver and kidney weights were the same in both sexes. Adult male offspring exposed prenatally to MCS and fed an HFD for 2 weeks before sacrifice had a higher absolute (but not relative) liver weight than did the sex-/diet-matched, air-exposed controls. We observed sex differences in relative liver weights, with those of the air-exposed female offspring greater than those of their exposure-matched male counterparts. Alternatively, relative liver weights in the prenatally smoke-exposed female group were lower than those of their air-exposed counter parts. In addition, some organ weights differed between the sexes in HFD-fed offspring, with absolute heart and kidney weights (in both exposure groups) being lower in female pups than in the age-/exposure-matched male offspring.

### Lipid parameters in the dams and offspring

**Figure 2:**
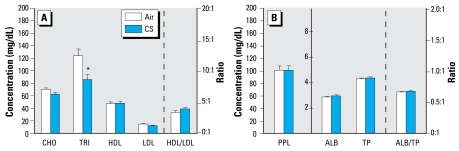
Effects of MCS exposure on plasma levels of CHO, TRI, HDL cholesterol, and LDL cholesterol and HDL/LDL ratio (*A*), and on levels of PPL, ALB, and TP, and ALB/TP ratio (*B*) in the dams. Values represent the mean (*n* = 8 dams/exposure regime) ± SE. Note the different scale in (*B*) for PPL compared with ALB and TP. *Significantly different (*p* < 0.05) from air-exposed dams.

We determined plasma levels of CHO, TRI, HDL, and LDL, as well as HDL/LDL ratios from pregnant mice on GD18 (Figure 2A). We also determined levels of PPL, ALB, and TP, along with ALB/TP ratios in the same mice (Figure 2B). Among these parameters, only TRI levels were significantly altered (decreased by 31%) in the dams after direct inhalation of MCS (compared with the air-exposed counterparts).

**Figure 3:**
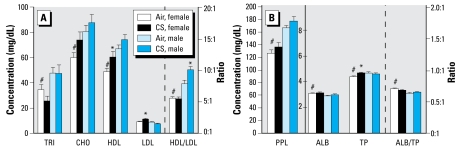
Effects of prenatal MCS exposure on plasma levels of CHO, TRI, HDL cholesterol, and LDL cholesterol and HDL/LDL ratios (*A*), and on levels of PPL, ALB, and TP and ALB/TP ratio (*B*) in 13- to 14-week-old female and male offspring fed a normal diet. Values represent the mean (*n* = 12–15 pups/sex/exposure regime) ± SE. Note the different scale in (*B*) for PPL compared with ALB and TP. *Significantly different (*p* < 0.05) from sex-matched, air-exposed offspring. ^#^Significantly different (*p* < 0.05) from exposure-matched male counterparts.

Adult male offspring prenatally exposed to MCS and fed a normal diet until sacrifice demonstrated a slight increase in HDL and a slight decrease in LDL levels (insignificant in both cases) compared with pups born to nonsmoking dams (Figure 3A) that resulted in a significant increase in the HDL/LDL ratio. In contrast to the modest effects seen in the male offspring, *in utero* MCS-exposed female offspring fed a normal diet after weaning demonstrated significant increases in plasma HDL, LDL, and TP levels compared with their sex-matched, air-exposed counterparts (Figure 3).

Baseline lipid parameters in mice fed a normal diet differed between the sexes. Levels of CHO, TRI, PPL, HDL, and TP, as well as the HDL/LDL ratio, were greater in air-exposed 13- to 14-week-old male offspring than in their exposure-matched female counterparts (Figure 3). Alternatively, baseline ALB levels and the ALB/TP ratio were higher in the female air-exposed pups than in their exposure-matched male counterparts.

**Figure 4:**
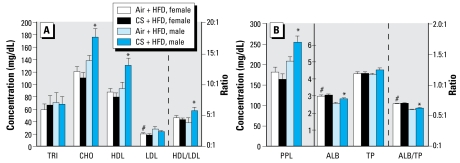
Effects of prenatal MCS exposure on plasma levels of CHO, TRI, HDL cholesterol, and LDL cholesterol and HDL/LDL ratio (*A*), and on levels of PPL, ALB, and TP and ALB/TP ratio (*B*) in 13- to 14-week-old female and male offspring fed an HFD. Values represent the mean (*n* = 6–8 pups/sex/exposure regime) ± SE. Note the different scale in (*B*) for PPL compared with ALB and TP. *Significantly different (*p* < 0.05) from sex-matched, air-exposed offspring. ^#^Significantly different (*p* < 0.05) from exposure-matched male counterparts.

Exposure to MCS *in utero* followed subsequently by a switch from a normal diet to an HFD in the adult offspring for 2 weeks before sacrifice resulted in dramatic differences between the sexes. A number of lipid parameters (i.e., CHO, HDL, PPL, and ALB levels), along with the HDL/LDL and ALB/TP ratios, were altered (compared with their air-exposed counterparts) in the male offspring fed an HFD (Figure 4). Interestingly, effects of pre natal MCS exposure observed in female offspring fed a normal diet were no longer visible in the HFD-fed group. These observations revealed that both male and female offspring exposed to MCS *in utero* exhibited dyslipidemia on a normal diet. Consumption of an HFD worsened the MCS-induced effects on CHO and HDL levels in the male, but not the female offspring.

### NMR analysis

We determined particle numbers and sizes of HDL, LDL, and VLDL subclasses in plasma samples from 13- to 14-week-old male and female offspring fed a normal diet. NMR analyses demonstrated that prenatal MCS exposure significantly increased total (air vs. MCS, 5.9 ± 5.9 vs. 56.6 ± 3.0 nmol/L; mean ± SE) and large (4.3 ± 4.3 vs. 42.4 ± 4.2 nmol/L) LDL particle concentrations in the adult female offspring. However, MCS exposure *in utero* had no effect on HDL (air, 20.2 ± 1.2 μmol/L) or “VLDL and chylomicron” (3.9 ± 3.9 nmol/L) particle concentrations in these same offspring; mean particle size of HDL, LDL, and VLDL was unaffected by exposure, with all values similar to that of the air control (9.0, 22.7, and 45.4 nm, respectively). In the male offspring, we observed no differences in HDL (air, 25.7 ± 1.9 μmol/L), LDL (48.9 ± 12.3 nmol/L), or VLDL (6.6 ± 3.1 nmol/L) particle concentrations or size (9.1, 22.3, and 45.8 nm, respectively) between treatment groups, despite a significant increase in HDL cholesterol level (air vs. MCS = 45.5 ± 3.2 vs. 63.6 ± 4.0 mg/dL) as estimated by NMR LipoProfile algorithm.

### TAC

**Figure 5:**
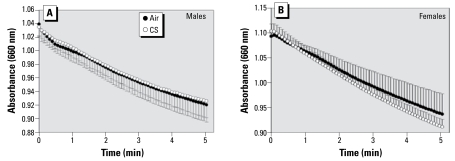
Effects of prenatal MCS exposure on TAC in 13- to 14-week-old male (*A*) and female (*B*) offspring fed a normal diet. Values represent the mean (*n* = 6 pups/sex/exposure regime) ± SE.

We determined TAC from plasma samples recovered from adult offspring of both exposure groups fed a normal diet (Figure 5A). No significant differences were observed between air- and smoke-exposed groups in either sex. Trolox equivalents calculated for the final time point (i.e., 5 min) were 0.55 ± 0.05 mM and 0.53 ± 0.06 mM for air- and MCS-exposed males, respectively; equivalents for air- and MCS-exposed female offspring were 0.64 ± 0.08 mM and 0.70 ± 0.04 mM, respectively.

## Discussion

The major findings of this study are that pre-natal exposure to MCS results in a significant sex-dependent increase in BW and changes in plasma lipids in the adult offspring. In female pups, the increase was evident on a normal diet, whereas the male offspring gained more weight on an HFD. These data also show that, compared with offspring of dams exposed to air alone, female offspring of dams exposed to MCS had higher levels of plasma HDL and LDL, whereas males showed higher cholesterol levels on an HFD. To the best of our knowledge, these data provide, for the first time, direct experimental evidence supporting the notion that prenatal exposure to MCS affects offspring weight gain and induces a lipid profile that could alter the offspring’s risk of CVD later in life.

Many adult illnesses have their origins in fetal and early childhood environment (Dietert and Dietert 2008; Fowden et al. 2005; Gluckman et al. 2008). Fetal/perinatal exposure to an environmental stressor, such as maternal malnutrition, hypertension, or cigarette smoking, appears to increase the risk of developing CVD later in life (Barker 2000; Cohen 2004; Louey and Thornburg 2005). It is believed that the prenatal environment can modify postnatal physiology through a process known as “programming,” which involves “adaptive changes in gene expression patterns that occur in response” to the stressor and that these alterations can “lead to altered growth of specific organs during their most critical times of development” (Louey and Thornburg 2005; see also Cohen 2004). Programming is evident in a number of risk factors that are only now becoming understood, including structural and cellular changes in the heart and coronary blood vessels, impaired endothelial function, and altered lipid metabolism (Louey and Thornburg 2005). In many cases, low birth weight seems to underlie the increased risk of athero sclerosis, hypertension, and/or metabolic syndrome in the adult, and this association led to the “fetal origins hypothesis” (Barker et al. 1989). For example, in a study comparing the frequency of ApoB mutations and lipid composition in full-term newborns, Akisu et al. (2004) demonstrated that CHO and ApoB concentrations in infants with restricted fetal growth were significantly higher than in normal-weight infants, suggesting that such dys lipidemia was associated with an adverse fetal environment.

Previous epidemiologic and toxicologic studies have reported that adolescent body mass index and prevalence of obesity are greater in offspring whose mothers smoked or were exposed to nicotine while pregnant (Al Mamun et al. 2006; Gao et al. 2005). Although a variety of confounding variables were accounted for, the contribution of unhealthy lifestyle choice and nutritional habits of children of mothers who continue to smoke during pregnancy could not be entirely isolated. Thus, even though exposure of mice up until birth only reflects exposure in humans for the first two trimesters, findings of our controlled experimental study provide direct evidence of an under lying biological mechanism (likely activated by *in utero* tobacco smoke exposure) that could contribute to and/or enhance future risk of excess weight in the exposed offspring. Our data suggest a strong sex dependence associated with observed effects, revealing a greater vulnerability of female offspring to weight gain later in life. In males, weight gain, apparent only in offspring fed an HFD, suggests inherent vulnerability that becomes apparent only in the face of excessive dietary fat (“second hit”). Although several hypotheses have been proposed to explain this relationship, including nicotine-mediated effects on hypothalamus development *in utero* and appetite control (Kane et al. 2000; Li et al. 2000; Slotkin 1998), the specific underlying mechanism(s) remains unknown.

Results of this study also demonstrate that fetal exposure to MCS is associated with a sex-dependent dyslipidemic profile in the adult offspring. Smoking is a leading cause of atherosclerosis acting through a wide spectrum of mechanisms, of which increased proatherogenic effects of dyslipidemia are critical (Zaratin et al. 2004). CS contains numerous oxidants and free radicals that can initiate and/or promote oxidative damage (Link et al. 2007). In humans, most cholesterol is synthesized in the liver and transported by LDL in the circulation. Macrophages are converted into foam cells by taking up oxidized LDL by the scavenger receptor SR-A or CD36 (Link et al. 2007; Ross 1993). The appearance of lipid droplet-filled foam cells in the subintimal space has been considered an early-stage atherosclerotic lesion or “fatty streak” (Ueyama et al. 1998). Increased plasma LDL levels present in the prenatally MCS-exposed female offspring reflect a proatherogenic phenotype where an elevation of LDL can lead to enhanced LDL uptake and oxidation in the blood vessel wall that could lead (ultimately) to the initiation and progression of atherosclerotic plaques (Glass and Witzum 2001).

In contrast to LDL, HDL inhibits the oxidative modification of LDL through anti-oxidative enzymes, including ApoA1 (Link et al. 2007). HDL cholesterol has also been shown to inhibit the infiltration of oxidized LDL into the vessel wall and stimulate cholesterol efflux from the foam cells back to the liver either for excretion in bile or for recycling [i.e., reverse cholesterol transport (Ueyama et al. 1998)]. Notable in our present study was an elevation in plasma HDL levels in pre-natally exposed female offspring and in the HDL/LDL ratio in exposure-matched male mice. Results from NMR analyses indicated that although we observed no change between the exposure groups in HDL particle concentrations [likely because of a small sample size (*n* = 3)], male offspring prenatally exposed to MCS (compared with sex-matched air controls) had higher plasma HDL levels. Although increased HDL levels generally indicate protection and decreased CVD risk, such changes can also act to bring about adverse outcomes, including disruption in critical cholesterol trafficking, potentially leading to long-term dyslipidemia; disruption in the cholesterol efflux pathway, in which HDL scavenges cholesterol for elimination from the body; and/or an increase in HDL of the proinflammatory type (Ansell et al. 2007; Zaratin et al. 2004). Furthermore, it has recently been reported that the atheroprotective capacity of HDL depends only partly on concentration and perhaps more importantly on its functionality (Sviridov et al. 2008). In line with this finding, we observed that the smoke-induced increase in offspring HDL level was not associated with any changes in plasma antioxidant function (i.e., TAC), suggesting that the increased HDL component was of a reduced “functional” capacity. Such HDL dysfunction was also observed by Navab et al. (2001), who reported that plasma HDL (prepared by fast-performance liquid chromatography) recovered from mice exposed to second-hand CS had a lower ability *in vitro* to prevent LDL oxidation. Dysfunction of HDL has also been associated with certain types of metabolic diseases. For example, Watson et al. (2007) showed that not only were high HDL levels not protective against coronary heart disease events in subjects with metabolic syndrome, but also that high HDL cholesterol levels were paradoxically associated with increased coronary heart disease events that could be due to inflammation-mediated changes. Thus, an increased level of HDL as observed in this study may not (necessarily) be protective or lead to a decreased risk of CVD.

Epidemiologic data suggest that children of mothers who smoked while pregnant maintain a dyslipidemic phenotype in early adulthood (I can et al. 1997; Jaddoe et al. 2008). For example, in a study that examined the relationship between maternal smoking during pregnancy and serum lipid, lipoprotein, and apolipoprotein levels in newborns (38 smoker and 42 non smoker mothers), babies of mothers who smoked during pregnancy showed significantly higher LDL/HDL ratios, along with CHO/HDL ratios (I can et al. 1997). In a prospective cohort study in which cholesterol levels were measured annually from 1975 to 1993 and again in 2002 (350 subjects 5–19 years of age at baseline), Jaddoe et al. (2008) observed a significantly higher annual increase in CHO and tendencies of higher LDL cholesterol and lower HDL cholesterol in children from smoking mothers (compared with those from nonsmokers). Data from animal studies support the epidemiologic findings. For example, 9-week-old male offspring born to pregnant spontaneously hypertensive rats exposed to nicotine via subcutaneous osmotic pumps throughout gestation had higher serum cholesterol levels than did age-matched controls (Pausová et al. 2003).

CS and some of its individual constituents have been shown to alter serum cholesterol levels in actively and passively exposed individuals (Barcin et al. 2006; Moskowitz et al. 1990). For example, in a study evaluating the effects of smoking (≤ 20 cigarettes/day) on serum lipids, Barcin et al. (2006) demonstrated that HDL level decreased in a stepwise fashion as the level of smoking increased in young healthy men. Also, Moskowitz et al. (1990) observed signifi cantly lower HDL levels in preadolescent children exposed passively to environmental tobacco smoke in families with at least one smoking parent (compared with children of similar age from nonsmoking families). Similar dys lipidemic effects have also been seen in rodent models exposed to CS constituents, such as nicotine and BaP. Ashakumary and Vijayammal (1997) demonstrated significant increases in CHO, PPL, and TRI, as well as in the amount of lipids associated with LDL and VLDL, in the sera of 6- to 7-week-old Sprague-Dawley rats treated with nicotine (3.5 mg/kg BW) for 90 days. In addition, 6- to 8-week-old ApoE-null mice treated by gavage with a single oral dose of 5 mg/kg BW BaP demonstrated lower serum LDL and higher HDL levels than did controls (Godschalk et al. 2003). The authors believed that the effects of BaP on serum lipid levels were due to the induction of hepatic cytochrome P450 enzymes and that, depending on the changes in HDL functionality, the observed increase in HDL was not necessarily beneficial. Taken together, the aforementioned studies suggest that some CS constituents may be transported across the placenta to the fetus, leading ultimately to dyslipidemia later in life.

Our findings support the link between maternal smoking and offspring dyslipidemia and demonstrate for the first time that exposure to MCS, at a dose range similar to human exposures, alters blood lipids in the offspring with only minimal effects on the directly exposed pregnant mothers. These findings under score the increased vulnerability of the developing fetus and suggest that prenatal insults during critical stages of fetal development can significantly influence later-life health risk.

In the present study, protein content differed between the diets; therefore, it is possible that some of the observed changes in lipid metabolism and/or BW may have been because mice are susceptible to small changes in calorie and/or protein intake. Future studies will employ HFD supplemented with protein (to assure equal protein intake between the groups) to determine the extent to which these dietary differences may have contributed to the observed differences.

In conclusion, the present study has demonstrated that exposure of mice to MCS during pregnancy can produce sex-dependent effects on two key risk factors of CVD in the adult offspring. The exact mechanism(s) by which prenatal exposure to MCS could have given rise to the observed changes in exposed offspring is currently unknown. Thus, additional studies are required to delineate specific mechanisms that may regulate cholesterol metabolism and its sensitivity to tobacco smoke constituents. Taken together, these findings encourage more intensified efforts to support smoking cessation programs during pregnancy and raise the possibility that maternal exposure to other environmental pollutants during fetal development could act to increase CVD risk of offspring later in life.

## Figures and Tables

**Table 1:** Effects of MCS exposure on body and organ weights of pregnant dams sacrificed on GD18.

	Exposure group^a^	
Body/organ (g)	Air	MCS
Body	33.50 ± 1.38	32.11 ± 1.21
Heart		
Absolute	0.11 ± 0.00	0.11 ± 0.00
Relative^b^	3.33 ± 0.10	3.40 ± 0.12
Kidneys		
Absolute	0.31 ± 0.01	0.30 ± 0.01
Relative^b^	9.32 ± 0.24	9.43 ± 0.26
Liver		
Absolute	1.69 ± 0.06	1.65 ± 0.04
Relative^c^	5.08 ± 0.20	5.16 ± 0.11
Lungs		
Absolute	0.17 ± 0.00	0.16 ± 0.00
Relative^b^	4.96 ± 0.12	5.08 ± 0.21

a Values represent the mean (*n* = 8 dams/exposure regime) ± SE.

b Relative organ weight = (absolute organ weight ÷ BW) × 1,000.

c Relative organ weight = (absolute organ weight ÷ BW) × 100.

**Table 2:** Effects of *in utero* MCS exposure on organ weights of adult male and female offspring fed either a normal diet or HFD.

		Exposure group^a^			
		Male		Female	
Diet	Organ (g)	Air	MCS	Air	MCS
Normal^b^	Heart				
	Absolute	0.14 ± 0.00	0.13 ± 0.01	0.11 ± 0.00#	0.12 ± 0.00#
	Relative^c^	4.55 ± 0.06	4.45 ± 0.20	5.26 ± 0.14#	5.06 ± 0.11#
	Liver				
	Absolute	1.30 ± 0.06	1.33 ± 0.08	0.90 ± 0.04#	0.99 ± 0.05#
	Relative^d^	4.34 ± 0.10	4.48 ± 0.09	4.34 ± 0.07	4.32 ± 0.14
	Kidneys				
	Absolute	0.49 ± 0.03	0.45 ± 0.02	0.31 ± 0.01#	0.32 ± 0.01#
	Relative^d^	1.64 ± 0.06	1.54 ± 0.06	1.50 ± 0.04	1.40 ± 0.05
HFD^e^	Heart				
	Absolute	0.14 ± 0.00	0.15 ± 0.00	0.12 ± 0.00#	0.12 ± 0.00#
	Relative^c^	4.33 ± 0.21	4.12 ± 0.17	4.93 ± 0.25#	4.50 ± 0.15
	Liver				
	Absolute	1.35 ± 0.05	1.58 ± 0.07*	1.28 ± 0.04	1.21 ± 0.07#
	Relative^d^	4.25 ± 0.08	4.40 ± 0.17	5.19 ± 0.13#	4.67 ± 0.13*
	Kidneys				
	Absolute	0.46 ± 0.02	0.49 ± 0.02	0.33 ± 0.01#	0.32 ± 0.01#
	Relative^d^	1.47 ± 0.08	1.38 ± 0.07	1.33 ± 0.04	1.25 ± 0.06

a Values represent the mean (*n* = 7–8 pups/sex/exposure regime/diet group) ± SE.

b Mice were fed a normal diet and sacrificed at 13–14 weeks of age.

c Relative organ weight = (absolute organ weight ÷ BW) × 1,000.

d Relative organ weight = (absolute organ weight ÷ BW) × 100.

e Mice were fed an HFD for 2 weeks before sacrifice at 13–14 weeks of age.

* Significantly different (*p* < 0.05) from the sex-/diet-matched, air-exposed control.

# Significantly different (*p* < 0.05) from the diet-/exposure-matched male offspring.
